# Network-oriented neurorehabilitation after stroke: a paradigmatic approach

**DOI:** 10.1186/s12984-026-02052-0

**Published:** 2026-08-18

**Authors:** Fabio M. Conti, Roger Gassert, Naz Doganci, Adrian G. Guggisberg

**Affiliations:** 1 Cumiasca, Switzerland; 2 Rehabilitation Engineering Laboratory, Department of Health Sciences and Technology, ETH Zurich, Zurich, Switzerland; 3 Division of Neurorehabilitation, Department of Clinical Neuroscience, University Hospital of Geneva, Geneva, Switzerland

**Keywords:** Stroke rehabilitation, Network neuroscience, Neuroplasticity, Personalized neurorehabilitation, Technology-assisted neurorehabilitation

## Abstract

Stroke recovery increasingly demands a reconceptualization of the brain as a dynamic network system. This opinion paper advocates for a network-oriented neurorehabilitation paradigm, grounded in the understanding that functional recovery relies on the reorganization of distributed, interconnected neural systems across the central nervous system. While traditional rehabilitation approaches often target isolated impairments, a broader challenge is in the insufficient specification and documentation of therapeutic processes and their underlying mechanisms, which are often incompletely described and difficult to systematically replicate or compare across studies. We argue that effective therapy must integrate cognitive, motor, sensory, as well as perceptual, emotional, and motivational processes through strategically designed, goal-directed exercises that activate residual plastic hubs and promote adaptive connectivity. From this perspective, rehabilitation becomes a process of guided network modulation, in which task demands are dynamically adjusted to the patient’s evolving capacities in order to optimize engagement and learning. Importantly, this approach responds to the need for clearer identification of treatment components, mechanisms of action, and active ingredients, enabling a more transparent and theoretically grounded description of therapeutic interventions. To illustrate these principles, we highlight key features of Cognitive Multisensory Rehabilitation (CMR) and its subsequent development into the Comparison of Actions (CTA) approach, which emphasizes action simulation and comparison within a network-oriented framework. Building on this rationale, we present a Model Structure for Network-Based Therapeutic Exercises, consisting of a five-phase model derived from a network-oriented interpretation of the CMR/CTA framework. This model provides an operational structure for translating theoretical principles into clinical practice, while making the underlying cognitive and sensorimotor processes of therapeutic action explicitly identifiable and parametrically modulable, thereby supporting greater clarity, reproducibility, and consistency in therapeutic design and implementation. In addition, we discuss how emerging technologies, such as robotics and virtual reality, may further support network engagement by enabling the controlled delivery, monitoring, and adaptation of these processes within structured therapeutic frameworks. By aligning therapeutic planning with network neuroscience, this model offers a structured yet flexible approach to personalized rehabilitation. It allows therapeutic tasks to be systematically adapted across distinct phases based on the patient’s neuropsychological profile, sensorimotor status, and functional goals, while making treatment components and mechanisms more explicit and clinically interpretable. In doing so, it supports a more individualized, functionally relevant, and theoretically grounded approach to post-stroke neurorehabilitation.

## Time to shift the paradigm

Stroke is increasingly understood not as a focal lesion alone, but as a disorder of brain networks, that is, distributed and dynamically interacting sets of neural regions whose coordinated activity supports cognitive, sensory, and motor processes, affecting both structural connectivity (the physical white matter pathways linking brain regions) and functional connectivity (the temporal coordination of activity between regions) [1]. This shift in perspective presents a compelling need to rethink rehabilitation models. The damage caused by stroke involves not only localized tissue loss but also a disruption of broader network architecture, which impairs the dynamic interactions essential for integrated sensorimotor, cognitive, and perceptual functioning.

In this context, network plasticity emerges as a fundamental concept: the capacity of the central nervous system (i.e., the brain and spinal cord) to reorganize and form new connections, not only in proximity to the lesion but across distributed systems, a process often referred to as network reorganization, encompassing adaptive and maladaptive changes in connectivity patterns [2, 3]. While the present work primarily focuses on brain networks, it is important to acknowledge that plasticity extends beyond the brain to include spinal circuits, which actively contribute to motor learning and recovery processes. For example, recent evidence has demonstrated intrinsic spinal cord plasticity and its dynamic interaction with supraspinal networks (i.e., neural systems located above the spinal cord, primarily within the brain) during motor skill acquisition [4], a perspective increasingly reflected in contemporary neurorehabilitation frameworks that emphasize the distributed nature of plasticity across the central nervous system [5].

A network-oriented approach to rehabilitation [6], that is, a paradigm that explicitly targets the restoration and modulation of interactions within and across neural networks, focuses on supporting this reorganization, facilitating local and global network integration (i.e., coordinated activity within and between brain systems), interhemispheric communication (i.e., interactions between the two cerebral hemispheres), and multisensory integration (i.e., the combination of information from different sensory modalities). It implies moving beyond isolated motor training to designing interventions that engage the nervous system’s large-scale architecture in meaningful and coordinated ways.

Yet, despite this growing understanding, most neurorehabilitation practices continue to rely on paradigms that insufficiently consider the complexity and dynamics of brain networks. The challenge is to bring neurorehabilitation in line with contemporary neuroscience, developing methods and tools that are explicitly structured to promote network reorganization.

This paper outlines the foundational principles of network-oriented neurorehabilitation and proposes a conceptual and practical framework for implementation. Central to this framework is a theoretical shift: functional recovery is seen as a result of network reorganization.

The approach emphasizes integrative therapeutic strategies that engage cognitive processes, understood as the set of mental operations enabling the acquisition, processing, and use of information (e.g., attention, memory, executive functions), together with sensory and motor domains through goal-directed activities (i.e., purposeful behaviors oriented toward achieving a specific outcome in a given context). In this context, it is important to distinguish between abilities and function: abilities refer to underlying capacities measurable under controlled conditions, whereas function reflects the effective performance of meaningful activities in real-world contexts, shaped by environmental and personal factors, consistent with the distinction between capacity and performance described in the International Classification of Functioning, Disability and Health (ICF). In this framework, we use the term “action” to denote a context-specific, goal-directed, meaningful behavior (as opposed to mere movement) emerging from the integration of multiple processes at a given moment. This notion is distinct from that of skill: while actions refer to situated instances of behavior, skills are learned and progressively refined capabilities that enable the efficient and consistent execution of actions across situations.

It advocates for a personalized and adaptive approach, tailored to each patient’s evolving neurological profile, as well as to their individual needs, values, and perspectives, which are essential for engagement in the therapeutic process. Exercises are structured to activate key network hubs (i.e., highly connected and functionally influential regions within brain networks) and to modulate inter-regional connectivity. Finally, the framework incorporates enabling technologies, such as robotics, virtual reality, and serious games, to enhance, sustain, and individualize network engagement, while recognizing that their effectiveness depends on active patient participation, motivation, and alignment with personally meaningful goals.

Within this broader context, we highlight key elements of the Cognitive Multisensory Rehabilitation (CMR) (see Sect. ”Cognitive Multisensory Rehabilitation (CMR) as a Network-Oriented Therapy Approach”) that exemplify several core principles of a network-oriented approach. While it is not the only path forward, CMR illustrates how therapy can be structured to activate distributed networks, engage reflective and motivational processes, and integrate clinical reasoning within a collaborative process that actively involves the patient, supporting their autonomy and incorporating their priorities into individualized therapeutic planning.

Our goal is not to advocate for one model over another, but to underline the necessity of a shared framework grounded in neuroscience and operationalized through diverse yet coherent therapeutic tools. This framework enables stroke rehabilitation to fully embrace the logic of brain networks.

## Foundational premise: motor function and cognition share common neural networks

The traditional distinction of cognitive and motor processes in neuroscience has been increasingly challenged by evidence revealing their deep interdependence. Large-scale brain networks are not strictly domain-specific, but multifunctional and dynamically interact depending on task demands [7, 8]. This flexibility suggests that some networks may contain core computational components that support both motor and higher-order cognitive functions [9, 10]. While this conceptual framework is well established in the literature (e.g., [11]), the present work builds upon it by translating these principles into a structured and operational framework for the design of individualized therapeutic interventions.

Motor control relies on internal representations that guide planning and execution, and these representations can be maintained offline and repurposed for cognition [10, 12]. Neuroimaging studies show that regions implicated in motor execution, including the primary motor cortex (M1), posterior parietal cortex (PPC), and premotor cortex, are also recruited during tasks that do not result in action execution, such as motor imagery and mental rotation [13–16] In addition, neuromodulation studies showed that disruption of M1 impairs mental rotation performance [17, 18]. Stimulation of the PPC can also evoke the intention or sensation of movement in the absence of execution, pointing to its role in internal action representation [19, 20]. A recent lesion-symptom mapping study also demonstrated that lesions in M1 and frontoparietal regions predict deficits in the mental rotation of both bodily and non-bodily stimuli [21]. Together, these findings support the idea that motor and cognitive functions rely on overlapping neural architecture.

While one might argue that mental rotation and motor imagery are still motor-related functions, converging evidence suggests that motor and frontoparietal regions are involved in non-motor cognitive domains as well. For instance, motor areas have been found to be engaged not only during action-related language processing but also in abstract and metaphorical language comprehension, suggesting compensatory or integrative functions [22]. Similarly, a recent study demonstrated that parietal and premotor regions that were identified through an unrelated finger movement task showed resting-state connectivity patterns predictive of working memory performance across verbal and spatial modalities [23]. Crucially, this suggests that frontoparietal regions that are implicated in motor planning contribute to higher-order cognitive processing even in tasks with no explicit motor demands [10]. Notably, this role is not limited to frontoparietal regions, as evidence also highlights the involvement of other structures, such as the cerebellum, in implicit learning and cognitive functions [24, 25].

Altogether, these findings challenge the conventional separation between motor and cognitive systems, instead pointing to a shared, network-level architecture where overlapping regions support both domains. This integrated view is particularly relevant for stroke patients, where damage to distributed networks may affect motor and cognitive abilities at the same time. For instance, cognitive deficits such as hemispatial neglect may significantly constrain the effectiveness of motor training [26]. The relationship between motor and cognitive impairments is not uniform: severe motor limitations do not necessarily entail reduced cognitive engagement. Instead, outcomes depend on the specific networks affected, as well as on individual patterns of reorganization and prior learning [27–29]. Acknowledging this variability supports the development of combined therapeutic strategies that can be flexibly adapted to the patient’s profile, rather than assuming a fixed coupling between domains.

## Network plasticity as key to functional recovery after stroke

Optimizing rehabilitation outcomes after stroke requires fully harnessing the central nervous system’s plastic potential. Mounting evidence shows that functional recovery is fundamentally driven by dynamic reorganization processes within structural and functional brain networks. Several reviews have previously summarized the principles of network plasticity [1, 2], which serve as a conceptual foundation for targeted therapeutic strategies. Although most of this work has focused on large-scale brain networks, converging evidence indicates that plasticity is a distributed property of the central nervous system, encompassing also brainstem and spinal circuits that interact with supraspinal networks during motor learning and recovery (e.g., [4, 5]).

Recovery is not determined solely by the extent of local tissue damage, but rather by the brain’s capacity to reorganize widespread neural circuits. However, when central motor pathways such as the corticospinal tract are severely compromised, recovery potential is substantially reduced, even in the context of network-oriented rehabilitation strategies [30–33].

Moreover, affected white matter tracts may undergo early-phase degeneration, which can propagate dysfunction to remote but interconnected brain regions, compromising the overall network architecture of both hemispheres [33–36]. Beyond these secondary degenerative processes, longitudinal studies have shown that stroke is also associated with widespread structural plasticity involving cortical hubs across both hemispheres, with both common and hemisphere-specific patterns that correlate with behavioral recovery [37]. This remote impairment is consistent with the classical concept of diaschisis as introduced by von Monakow in 1914 [38]—a functional depression of brain areas connected to the lesion site—, and aligns with contemporary reinterpretations that frame diaschisis within a network neuroscience perspective (i.e. connectional diaschisis) [39]. Clinically, such distributed effects are also reflected in the observation that the “less-affected” limb in hemiparetic patients is often not functionally normal when compared to age-matched controls, with deficits that vary depending on the hemisphere of damage and the severity of impairment [40]. Recent evidence further indicates that targeted rehabilitation of the ipsilesional arm can improve motor performance in chronic stroke patients, underscoring the functional relevance of these often-overlooked deficits [41]. These findings further support the notion that stroke-related dysfunction extends beyond focal lesions, affecting bilateral network organization and contributing to variability in behavioral outcomes.

Effective restoration of complex functions thus requires the recruitment and reconnection of distributed brain networks [2]. These adaptive processes extend beyond the lesion site to involve structurally intact, yet functionally connected, areas of the brain. In particular, preserved ipsilesional regions can assume compensatory roles through increased intrahemispheric connectivity [42–44]. Meanwhile, the contralesional hemisphere also undergoes structural and functional changes, though its impact on recovery is context-dependent, and can be either beneficial or maladaptive. Importantly, these effects depend not only on lesion characteristics but also on time after stroke, as interhemispheric interactions evolve dynamically across recovery stages and may reflect ongoing reorganization processes rather than a fixed imbalance [45].

Rehabilitation must therefore reflect the dynamic and distributed nature of post-stroke plasticity. The therapeutic goal is not simply to restore isolated motor or cognitive functions, but to promote the integrative reorganization of neural networks that jointly sustain movement, language, and cognition.

This requires interventions that address these domains in a coordinated, network-aware manner, recognizing their functional interdependence and the fact that they rely on overlapping neural substrates that share neuronal resources. Such an integrated strategy is essential for restoring not only complex problem-solving abilities, but also coherent mental representations and the embodied experience of initiating and controlling actions. More broadly, rehabilitation can be understood as the progressive recovery and refinement of a continuum of behaviors, ranging from basic sensorimotor responses to higher-level, learned skills that support efficient and contextually appropriate performance in everyday life. From this perspective, functional recovery involves the re-establishment of adaptive skills through practice, engaging distributed neural processes that are continuously shaped by experience—an interpretation consistent with the “heksor” framework proposed by Jonathan R. Wolpaw and colleagues [46], in which skills are conceptualized as dynamic, distributed neural substrates extending across the central nervous system.

This is particularly relevant for the affected limb, where the sense of ownership and voluntary control is often compromised, reflecting altered mechanisms of action monitoring and error processing that influence how the limb is used in everyday behavior. These alterations are typically more pronounced following right-hemisphere lesions [47–49], and more rarely observed in left-sided lesions [50]. Consistently, recent evidence suggests that hemispheric damage may differentially affect adaptive control of the limbs, with right-hemisphere stroke associated with reduced error detection and compensation, and consequently diminished adaptive use of the contralesional limb, whereas left-hemisphere stroke may preserve or even enhance these mechanisms [51].

## Network-based rehabilitation

### Cognitive multisensory rehabilitation (CMR) as a network-oriented therapy approach

Among the therapeutic approaches that align with a network-oriented view of rehabilitation [6, 52, 53], we consider the *Cognitive Multisensory Rehabilitation* (CMR) developed by Carlo Perfetti in the 1970s (*Esercizio Terapeutico Conoscitivo*) as a particularly compelling example. The following section illustrates how CMR embodies key principles of this framework, without excluding the possibility that aspects of these principles may be found in other rehabilitation approaches.

CMR conceptualizes motor recovery not as a pure motor process, but as a problem-solving activity that engages perception, attention, and cognition alongside motor execution. This cognitive perspective is central to its therapeutic rationale. While this section does not aim to present the method exhaustively, it highlights core features relevant to network-oriented strategies. The publication by Van de Winkel [107] provides an excellent and concise description of how typical CMR exercises are conducted. For a more in-depth study on the subject, readers are referred to the works of Carlo Perfetti cited in the references.

Building on the emerging body of knowledge on neuroplasticity, CMR conceptualized stroke rehabilitation as a cognitively mediated learning process. Here, “cognitive” refers to the structured engagement of conscious, goal-directed processes in the exploration and resolution of sensorimotor tasks. This does not exclude the contribution of implicit learning mechanisms, which operate in parallel and interact with explicit strategies during motor learning (e.g., [54]). Recovery is thus understood to arise from the interplay of these processes, supporting the reactivation and integration of distributed neural networks rather than local restitution alone [55–59]. In this sense, the reacquisition of function can be viewed as the progressive re-establishment of adaptive skills supported by distributed neural substrates, consistent with the “heksor” framework proposed by Jonathan R. Wolpaw and colleagues [46].

Translating these principles into practice, CMR-exercises follow a progression of increasing complexity. In first-level exercises, sensorimotor perception is facilitated through passive mobilization by the therapist. With visual input typically excluded, the patient is instructed to direct attention toward proprioceptive, tactile, and kinesthetic cues. This early perceptual focus is intended to optimize central processing without eliciting maladaptive excitation of motor circuits, a mechanism considered relevant for reducing diaschisis [55]. In second-level exercises, active movement is initiated by the patient under guided conditions, while third-level tasks involve autonomous motor execution following verbal instructions. Across all levels, the patient is consistently engaged in resolving a sensorimotor problem through bodily action, drawing on the same core afferent modalities. By “sensorimotor problem,” we refer to a task that requires the patient to actively explore and interpret sensory information through movement in order to achieve a specific perceptual and action-related goal, for example, discriminating between different distances or object properties (e.g., texture or shape) through hand or finger exploration typically involving the discrimination of task-relevant sensory properties (e.g., distance, texture, or shape) through finger exploration. The therapist functions as a dynamic mediator of attentional focus and perceptual discrimination, assisting in the decoding and refinement of the sensorimotor experience both during task execution and in retrospective analysis.

Overall, the CMR approach laid the foundational framework for a neurocognitive view of rehabilitation, shifting attention from peripheral muscle training to central motor organization. It emphasized intentional, goal-directed action shaped by multisensory integration and cognitive processing. While these concepts have been further developed and discussed in later network-based accounts of recovery (e.g., [11]), it is important to note that core principles of this perspective were already articulated within the CMR framework since its early formulation by Carlo Perfetti in the late 1970s, as previously outlined. More recent literature can therefore be seen as converging with, rather than originating, these neurocognitive assumptions.

In recent years, this framework has evolved into the “*Comparison of Actions*” (CTA) approach, which can be considered both a refinement and a natural progression of CMR [60, 61]. CTA emphasizes the mental simulation and structured comparison of meaningful actions to promote recovery. It engages a premotor-parietal network overlapping with regions activated during actual movement and motor imagery in stroke patients [13, 14, 62–64].

### Core network-relevant principles of the CMR approach

The selected principles presented below exemplify strategies that can be broadly generalized and integrated into therapeutic practices aligned with a network-oriented neurorehabilitation framework.

#### Motor imagery as a mechanism for internal network activation

Motor imagery was introduced into neurocognitive rehabilitation by Carlo Perfetti in 1987, bringing a new dimension to therapeutic engagement by emphasizing the role of internally generated action representations. Its clinical implications were further elaborated in the following years [65–67].

Within a neurocognitive perspective, cognition can be understood as the set of processes through which stored information is activated, manipulated, and transformed in order to guide goal-directed action. In this sense, motor imagery is conceptualized as a cognitive process involving the activation, manipulation, and transformation of both conscious and unconscious stored information [68–70].

Importantly, these processes do not operate in isolation but are modulated by attentional and motivational factors that influence how effectively action representations are constructed and used. In line with more recent accounts of motor learning, such as the OPTIMAL theory of motor learning, attention and intrinsic motivation contribute to strengthening the coupling between goals and actions, thereby facilitating learning and performance [71].

From this perspective, motor imagery does not merely involve the recall of previous motor patterns but enables the construction of new sensorimotor representations within a goal-oriented and motivationally shaped cognitive context, which may facilitate the development of more effective action strategies [72].

However, evidence shows that stroke survivors—particularly those with frontal or parietal lesions—may exhibit altered motor imagery performance, such as prolonged imagined movement times or reduced representational accuracy [73–77], as the fronto-parietal network plays a crucial role in the generation of motor imagery [78]. Findings from lesion-symptom mapping studies and meta-analyses further confirm that impairments in motor imagery vividness and temporal congruence are closely associated with lesions in the fronto-parietal network, basal ganglia, and premotor regions [79]. Complementary task-based fMRI and functional connectivity studies further demonstrate that motor imagery relies on dynamically coordinated activity across these regions, highlighting the importance of network-level integrity rather than isolated regional function [14, 78]. Given that stroke can differentially disrupt these network interactions, motor imagery capacity is likely to vary substantially between patients, underscoring the importance of assessing each patient’s capacity for motor imagery and using it in a tailored way (see Sect. ”Toward Personalized Network Rehabilitation”).

This perspective also supports a more personalized approach to neurorehabilitation, in which patients may differentially respond to motor imagery–based and network-oriented interventions depending on the integrity of the underlying neural substrates. In this context, future research should aim to identify responders and non-responders to such interventions.

For patients who show limited motor imagery ability or reduced responsiveness, alternative strategies may be required, including approaches that rely more directly on action execution, sensorimotor feedback, or other modalities of network engagement. This highlights the need for flexible, patient-specific therapeutic pathways rather than a one-size-fits-all application of motor imagery.

#### Language as a structuring mechanism for network coordination

Language plays a strategic role within the therapeutic framework of CMR, as verbal interaction encourages patients to articulate their perceptions (perceptions, understood here as the subjective interpretation of sensory information), sensations, and perceived errors. This reflective process enhances self-awareness, fosters insight, and reinforces learning.

Crucially, recent neuroscientific evidence suggests that these metacognitive processes—central to perception, error monitoring, and decision-making—are supported by interactions between prefrontal regions such as the dorsolateral and frontopolar cortex [80–82]. In this context, both overt and inner speech may function as mediators of metacognitive control, facilitating the dynamic reorganization of neural networks involved in sensorimotor integration when embedded in therapeutic exercises. Additionally, inner speech serves as a powerful cognitive tool that supports action planning and internal simulation. Internally generated language—such as self-directed speech and silent verbalization—actively contributes to the construction of motor intentions and the anticipation of sensorimotor outcomes. Evidence from research on inner speech and motor imagery [83, 84] indicates that motor sequences are often mentally rehearsed and structured through linguistic scaffolding, facilitating the organization and temporal coordination of physical movement. This internal linguistic mediation is essential in CMR approaches that emphasize the cognitive structuring of actions as a pathway toward restoring functional motor control.

These internal linguistic processes are typically implicit in nature, operating automatically to support thought organization and action planning. However, within neurorehabilitation frameworks such as CMR, they can also be shaped through explicit therapeutic intervention. Patients may be guided to consciously attend to specific aspects of their internal dialogue, restructuring it in ways that support more effective motor performance. For example, when a patient relies on inefficient or maladaptive movement strategies, the therapist can direct attention toward relevant sensory cues or task goals, thereby modifying the internal verbalization that guides action planning.

In this sense, inner speech represents a dynamic interface between implicit and explicit processes, which can be strategically engaged during rehabilitation depending on the patient’s neuropsychological profile, particularly their attentional capacities, information processing abilities, and decision-making functions.

This transition from implicit internal processes to their explicit modulation through therapeutic guidance provides the basis for strategic learning approaches, as discussed in the following section.

However, these strategies may be less effective in patients with impaired awareness of deficits, such as those presenting with Anosognosia, in whom the capacity for self-monitoring and error recognition is compromised. Importantly, this impairment is often partial and may affect the accessibility of internal monitoring signals to conscious reflection rather than their complete absence. In such cases, the use of language as a reflective tool may require adaptation and integration with other therapeutic strategies, including approaches that enhance engagement and awareness through behavioral and motivational modulation.

Similarly, in patients with Aphasia due to left-hemisphere lesions affecting language networks, the effectiveness of verbal interaction may be reduced at different levels, including comprehension, internal language processing, and verbal expression, depending on the specific profile and severity of the impairment. In these cases, therapeutic communication should be adapted to the patient’s residual abilities, using multimodal and supportive strategies to facilitate participation and engagement. This process often requires close interdisciplinary collaboration, particularly with speech and language therapists, in order to optimize the use of preserved communicative channels.

Nevertheless, when deficits in awareness or language are severe and persistent, they may represent significant limitations to the effectiveness of language-based strategies for structuring network coordination, highlighting the need for flexible and individualized therapeutic approaches.

#### Strategic learning as a mechanism for network adaptation

Strategic learning with explicit instructions can be particularly effective in a network-oriented practice strategy [85–87]. It allows targeted recruitment of networks involved in motor control and motor learning, especially when deficits are present. By consciously reflecting on movement sequences, e.g., in the context of motor imagery tasks, patients can develop and apply alternative movement strategies [88–91].

Motor deficits often impair automatic processes—habitual, low-attention routines that usually ensure fluid and efficient movement without conscious control [92, 93]. In early rehabilitation, strategic learning helps compensate for such impairments by enabling conscious control. Instructions enhance error perception and correction: patients learn to understand *why* a movement failed and *how* to adapt it. This explicit error-based learning enables transfer of movement knowledge to novel or changing conditions. Patients not only perform movements; they also analyze and adapt them, which supports long-term autonomy. While strategic learning dominates early stages, it is believed to prepare for a shift toward more implicit, success-based, goal-directed strategies essential for motor automatization [85, 87].

This progression is consistent with rehabilitation approaches that incorporate “transfer packages,” as originally proposed by Edward Taub within constraint-induced movement therapy, which emphasize problem-solving strategies, overcoming contextual barriers, and promoting self-management beyond the clinical setting. Such approaches have been shown to facilitate the transfer of learned skills to real-life situations and to support the gradual transition from explicit, cognitively guided strategies to more automatic and efficient motor behaviors [54].

As shown by Spampinato & Celnik [87], instruction-based learning can promote feedforward motor control by fostering the formation of internal predictive models. These mental representations help patients anticipate outcomes, reducing reliance on external sensory information and performance-related signals, which are often delayed or impaired post-stroke.

However, certain neuropsychological deficits limit the formation of such internal representations. Lesions in the parietal and prefrontal cortices—particularly the dorsolateral prefrontal cortex, crucial for conscious motor decisions—can hinder use of this strategy. In addition, damage resulting in disrupted dynamic interactions at the network level (i.e., frontoparietal network) can also lead to impaired top-down modulation, rendering certain strategies less accessible [94, 95]. Therapists must therefore consider cognitive impairments and adapt task complexity accordingly. The ability to engage in strategic learning should be assessed at the functional level, taking into account cognitive domains such as attention, executive functions, and comprehension abilities. Therapists must therefore consider the patient’s neuropsychological profile and adapt task complexity and instructional strategies accordingly. Moreover, this approach requires that patients are able, at least to some extent, to understand and use task-related instructions (see Sect. ”Toward Personalized Network Rehabilitation”).

### Evidence supporting CMR as a network-oriented therapy

#### Clinical trials

In their review of 2022, Maria del-Cuvillo-Yges and colleagues [96] provide a synthesis of studies published on CMR between 2012 and 2021, including research on stroke rehabilitation as well as interventions in non-neurological and pediatric populations. Approximately 85% of the included patient samples were neurologically affected, with stroke representing the most prevalent diagnosis (~ 80%).

Within this body of literature, several studies suggest that CMR may yield functional outcomes comparable to, or in some instances exceeding, those of conventional therapeutic approaches. Reported benefits include improvements in upper limb function, gait, balance [97], and aspects of quality of life in both neurological and orthopedic populations. Similar conclusions were drawn by an earlier meta-analysis conducted by Dominguez-Ferraz [98], which reviewed studies published between 1999 and 2014.

However, these findings should be interpreted in light of the methodological heterogeneity of the available evidence. The studies included in these reviews comprise randomized controlled trials, pilot studies, and case series, often with relatively small sample sizes and variable methodological quality. While some investigations, such as the assessor-blinded randomized study by Kim and Jang [97], provide controlled comparisons, many studies do not fully meet the criteria associated with higher-phase clinical trials, including detailed characterization of control interventions, blinding procedures, and explicit linkage between intervention components and their underlying mechanisms.

Therefore, although the current evidence is encouraging, it primarily supports associative rather than definitive cause–effect conclusions, highlighting the need for more rigorously designed studies to strengthen the clinical and translational validity of CMR.

The benefit of CMR has been less visible for motor assessments focusing on easily measurable variables such as muscle strength or range of motion. CMR-based interventions often target more integrative or qualitative aspects of behavior, such as improvements in body awareness, cognitive self-regulation, or functional autonomy. For example, an intervention that enhances body awareness can contribute to improved movement control and independence, despite yielding minimal change on conventional mobility scales [99–101].

In this context, an important open question concerns the durability and generalization of these gains over time and across functional situations. While some studies suggest potential transfer to everyday activities, the extent to which these improvements are maintained in the long term remains insufficiently established, highlighting the need for longitudinal studies with follow-up assessments.

The cognitive effects of network-oriented rehabilitation may be more apparent when evaluated through dedicated neuropsychological outcomes. A recent study by Huai et al. [102] reported that cognitive therapy enriched with sensorimotor components led to greater gains in cognitive recovery post-stroke than standard neuropsychological interventions alone. Importantly, these effects were detected using domain-specific measures of attention and executive function, suggesting that outcome sensitivity plays a critical role in capturing the effects of such interventions. Moreover, the study provided insight into potential mechanisms of action, showing that improvements were associated with changes in functional connectivity involving the dorsolateral prefrontal cortex and related cognitive networks. This highlights the importance of aligning intervention targets, underlying neural mechanisms, and outcome measures when evaluating network-oriented approaches, moving beyond a generic network-based framework toward a more precise characterization of how CMR may promote recovery.

These findings suggest that the interaction between sensorimotor stimulation and cognitive recovery is not uniform across patients, but instead varies depending on the individual’s pre-existing sensory, attentional, and cognitive capacities. While this interindividual variability is a common feature across rehabilitation interventions, it is often attenuated in controlled clinical trials due to strict inclusion criteria, which tend to produce more homogeneous samples and facilitate statistical comparisons, albeit at the expense of generalizability. From this perspective, the variability in response to integrative interventions such as CMR may be more pronounced in real-world clinical settings, where patients present with heterogeneous neuropsychological and sensorimotor profiles. This supports the argument that therapy outcomes are highly individualized: patients with specific sensory-motor or attentional characteristics may be more responsive to interventions that align with their residual network dynamics.

This aligns with accumulating evidence in stroke rehabilitation emphasizing interindividual variability in recovery trajectories [2, 103], and highlights the need for individualized, network-informed interventions—an approach strongly advocated by Chen et al. [104], who propose tailoring rehabilitation to neuroanatomical and biomarker-based patient characteristics. For example, a patient who develops greater body awareness through sensorimotor-cognitive stimulation may be better positioned to relearn meaningful motor strategies for daily activities. Although such gains may be less visible in the short term, they may support more sustainable long-term functional improvements.

The development and implementation of specialized, sensitive assessment tools is necessary to support more nuanced evaluation of such interventions. In particular, there is growing consensus on the importance of capturing the quality of movement through fine-grained measures, including kinematic and kinetic analyses, which allow a more precise characterization of motor control and recovery processes [105]. Such approaches are considered essential for distinguishing true motor recovery from compensatory strategies and for linking behavioral changes to underlying neural mechanisms.

Within this context, the clinical *Motor Evaluation Scale for Upper Extremity in Stroke Patients* (MESUPES) [106] distinguishes itself by evaluating the quality of arm and hand performance—such as fluidity, coordination, and the ability to adapt movements to specific motor tasks—during post-stroke recovery through the execution of purposeful movements, providing a more detailed assessment of functional motor control than measures focused solely on impairment or task success. Its clinical applicability and validity have been demonstrated in several stroke studies [101,107].

#### Imaging studies

Neuroimaging offers a promising avenue for investigating therapy-induced changes in brain network organization. By analyzing alterations in the interactions among sensory, motor, and cognitive systems following interventions such as CMR, imaging studies can provide insight into the plastic mechanisms that underlie behavioral recovery. This approach may help clarify the neurocognitive processes engaged by CMR and offer an objective framework for evaluating and refining therapeutic strategies. Several studies have begun to explore whether and how CMR influences functional brain connectivity in stroke patients. A notable contribution in this domain is the work by Van de Winckel and colleagues [107], which examined brain processes associated with sensorimotor recovery following CMR in post-stroke adults. Earlier neuroimaging studies conducted by the same lead authordemonstrated that somatosensory discrimination tasks characteristic of CMR engage distributed frontoparietal, insular, and cerebellar networks, includingthe parietal operculum [108–111] Building on these findings, subsequent work highlighted the involvement ofOP1/OP4 (SII), the insula, and cerebellar crus I/II during shape-discrimination tasks requiring focused sensory attention [107]. Importantly, activation of these regions appeared contingent on focused attention to the sensory task, indicating that attentional modulation may play a keyrole in facilitating network-level plasticity.

 This observation is consistent with theoretical accounts of motor learning, such as the OPTIMAL theory [71], which emphasize that attentional focus contributes to strengthening the coupling between goals and actions. Within this framework, attention can be understood as a critical factor not only for behavioral performance but also for shaping the efficiency of functional network engagement and plasticity during rehabilitation.

Based on these findings, the authors hypothesize that CMR may influence the multimodal parietofrontal network via OP1/OP4—an integrative hub that is often disrupted in stroke patients across different stages of recovery.

More specifically, they propose that CMR could enhance resting-state functional connectivity between OP1/OP4 and other cortical regions involved in sensorimotor integration. Their results support this hypothesis, showing increased functional connectivity between OP1/OP4 and regions targeted by CMR following the intervention. These findings suggest that CMR may contribute to network reorganization by modulating connectivity within a distributed, multimodal integration network.

If replicated and expanded, such connectivity changes could serve as candidate biomarkers for therapeutic responsiveness. Identifying neural correlates of recovery may enable more precise monitoring of intervention effects and support the development of individualized rehabilitation strategies, calibrated to a patient’s specific neuroplastic potential.

However, achieving this goal will require substantial efforts in data harmonization and longitudinal validation across studies. In this context, recent perspectives on AI-driven precision neurorehabilitation highlight the potential of integrating large-scale, multimodal datasets to build individualized patient models and test intervention strategies in a systematic way [112]. In particular, collaborative AI frameworks, where computational models support but do not replace clinical reasoning, have been proposed to generate patient-specific profiles and simulate treatment effects, thereby enhancing decision-making and optimizing intervention planning.

Complementary approaches based on reinforcement learning further suggest that adaptive, model-based systems could iteratively refine rehabilitation parameters, such as timing, intensity, and task selection, based on individual patient responses, moving toward dynamically personalized treatment strategies [113]. Together, these developments point toward a future in which biomarker-informed rehabilitation is integrated with data-driven modeling within learning health systems to support more precise, responsive, and individualized care.

### A model structure for network-based therapeutic exercises

The exercise structure proposed here represents a conceptual interpretation of the CMR framework, viewed through the lens of network-oriented neurorehabilitation. Rather than aiming to define the specific neural networks involved, this model highlights five observable and functionally distinct phases that can be identified in the therapeutic process. These phases serve as a heuristic scaffold for designing exercises that reflect the complexity of real-life actions and engage distributed cognitive and sensorimotor processes.

This reading identifies and abstracts the core components of CMR as an example of how rehabilitation exercises can be systematically designed to engage distributed neural networks. The resulting *five-phase model* highlights cognitive and network-level processes relevant to task-oriented therapy, offering a structure in which therapeutic tasks are purposefully constructed and parametrically modulated based on the patient’s evolving neuropsychological profile, sensorimotor status, and individualized goals. While inspired by the CMR approach, this model aims to provide a generalized and adaptable framework for designing rehabilitation programs consistent with the principles of network-based recovery.

#### Phase 1: task encoding

Patients receive verbal and gestural instructions, engaging language and multimodal integration networks. This phase involves language comprehension, gesture interpretation, and contextual processing, laying the groundwork for goal-directed action.

In patients with apraxia, particularly those with impaired gesture comprehension (e.g., ideational apraxia, ideomotor apraxia) [114] or with gesture agnosia, the ability to decode gestural instructions may be compromised. In addition, patients with aphasia [115] may also show reduced performance in gesture comprehension tasks, further affecting the processing of multimodal instructions. In such cases, task encoding relies more heavily on alternative input channels, including simplified verbal instructions, object-based demonstration, environmental cues, or physical guidance.

#### Phase 2: mental simulation and planning

Patients mentally simulate the required manual task as part of the action planning process, including elements of outcome anticipation, drawing on prior experiences and stored motor patterns.

The role of motor imagery as a mechanism for internal network activation has been introduced in Sect. ”Motor Imagery as a Mechanism for Internal Network Activation”. From the operational perspective of the present model, it is important to emphasize that action planning can be understood as relying on structured mental representations that organize goal-directed behavior across multiple levels of abstraction, from single motor elements to complex action sequences [116]. Already at the level of motor imagery, internal simulation contributes to the learning and structuring of sequential and context-dependent actions, extending its effects beyond the imagined components to influence the performance of sequentially coupled overt movements [117]. An outcome anticipation belongs to the process, actions are represented in terms of their anticipated sensory consequences, which guide both planning and execution processes [118–120].

Within this framework, mental simulation in the proposed model builds on the retrieval of stored motor patterns while extending beyond it into an active, goal-directed process that constructs, organizes, and refines action representations across levels of abstraction, shaped by prior experience, attentional modulation, motivational factors, and the broader network of action-related cognitive processes.

#### Phase 3: strategy selection and decision-making

Patients select a problem-solving strategy. Decision-making processes integrate motivational, cognitive, and affective inputs.

The selection of strategy draws on previously acquired strategies, including those developed through strategic learning processes (see Sect. ”Strategic Learning as a Mechanism for Network Adaptation”), and reflects the patient’s current cognitive, motor, and contextual constraints. From the operational perspective of the present model, strategy selection precedes and constrains decision-making processes, defining the space of possible actions. Within a selected strategy, decision-making mechanisms guide the selection and adaptation of specific actions in response to task contingencies.

These processes can be interpreted within neurocognitive models of decision-making, which describe the integration of cognitive, motivational, and affective inputs within distributed frontal–striatal networks, supporting value-based action selection (e.g., [121, 122]. From a network perspective, coordinated activity across prefrontal, striatal, and limbic regions enables the integration of internal goals, environmental constraints, and expected outcomes, consistent with the expected value of control framework [123].

In rehabilitation contexts, these processes are further modulated by attentional and motivational factors. As emphasized by the OPTIMAL theory of motor learning [71], intrinsic motivation and goal–action coupling enhance both performance and learning by strengthening the linkage between selected strategies and their behavioral implementation.

#### Phase 4: action execution

The movement is performed (or imagined, when guided by the therapist), requiring precise sensorimotor integration. All available sensory information at movement onset is integrated into the processes necessary to generate task-specific motor output. Depending on task demands, execution may rely on either explicit cognitive control or pre-established automatic motor routines.

The patient is confronted with a sensorimotor problem (see Sect. ”Cognitive Multisensory Rehabilitation (CMR) as a Network-Oriented Therapy Approach”) that requires the generation of goal-directed movement. Action execution (whether overt or, when guided by the therapist, imagined) depends on the capacity to produce task-specific motor output through precise sensorimotor integration.

At movement onset, all available sensory information is integrated with task goals and prior representations to support the generation of appropriate motor commands. This process is consistent with established models of sensorimotor control, in which afferent sensory signals are combined with predictive motor commands generated by internal models [124, 125]. Through this integration, the system is able to generate context-sensitive motor output and to support online control via continuous comparison between predicted and actual sensory feedback.

From a network perspective, motor execution and adaptation emerges from dynamic interactions within distributed sensorimotor systems rather than from isolated modules. These interactions enable flexible, non-linear integration of sensory inputs within cortical and subcortical regions, supporting adaptive mappings between sensory information and motor commands that are essential for skilled action [126, 127]. Ongoing error-based learning and neuroplastic mechanisms further refine this process, allowing continuous adaptation to environmental demands [128].

Within this framework, the degree of cognitive control involved in execution varies as a function of task demands and learning stage. Execution may rely on controlled processes requiring attentional resources or on automatized motor routines, consistent with dual-process accounts of skill acquisition [93]. This balance reflects the dynamic interaction between top-down control and more automatic sensorimotor processes, representing a key mechanism through which behavior remains both flexible and efficient.

In neurological populations such as stroke patients, the effectiveness of this process depends on the reorganization of large-scale brain networks rather than isolated regional recovery [27]. From a rehabilitation perspective, action execution is best understood as part of a continuous sensorimotor loop, in which ongoing movements generate sensory consequences that are immediately reintegrated to refine subsequent behavior [129].

In this sense, action execution does not represent the endpoint of the process but a dynamic interface through which predictions, decisions, and sensory feedback are continuously integrated.

#### Phase 5: reflection and adaptation

Patients and therapists jointly evaluate performance. This reflective process engages metacognitive networks, promotes insight, and guides the adjustment of future tasks. Emphasis is placed on reinforcing effective strategies and identifying areas for targeted improvement. In addition, this phase aligns with principle-based neurorehabilitation approaches such as the Accelerated Skill Acquisition Program (ASAP), which emphasize the integration of skill acquisition, capacity building, and motivational factors, including autonomy support and self-efficacy [130]. Within this perspective, therapist–patient interaction during performance evaluation represents a critical opportunity to leverage ‘teachable moments’, enhancing engagement and optimizing motor learning through individualized feedback and shared goal setting.

This structured model supports a network-based rehabilitation framework, where cognitive strategies such as language use, motor imagery, and decision-making are embedded across all phases. It also allows for the adaptation of performance demands to match the patients' current cognitive and motor abilities. In line with the Challenge Point Framework, optimal learning is expected when task difficulty is continuously adjusted to the individual’s skill level, thereby maximizing the informational value of practice and promoting both motor learning and motivation [131]. It may serve as a useful basis for developing individualized therapies, including those incorporating neurophysiological monitoring and feedback.

## Toward personalized network rehabilitation

Rehabilitation should engage residual capacities across all recovery phases, supporting the patient’s ability to act through tasks that integrate motivation, planning, execution, and reflection—without prematurely constraining plastic potential. Personalized network rehabilitation aims to adapt therapy to each patient’s unique clinical presentation, including lesion location, severity, and cognitive-motor profile. This perspective is consistent with principle-based approaches to neurorehabilitation that frame recovery as the result of interacting processes, including skill acquisition, capacity building, and motivational factors such as autonomy support, working together to shape meaningful, goal-directed behavior [130]. Importantly, beyond cognitive and motor processes, perceptual, emotional, and social domains also play a critical role in influencing engagement, motivation, and agency, and should be considered integral components of rehabilitation. From a network-oriented perspective, these factors dynamically interact with cognitive and sensorimotor systems to shape behavior across all phases of action.

Broader factors such as age, sex, comorbidities (e.g., diabetes, depression), genetic predisposition, motivation, and psychosocial context also influence outcomes and should inform therapy planning.

Within this framework, effective rehabilitation relies on aligning goals with actions in ways that enhance engagement and learning, supporting adaptive cycles of performance and recovery.

Personalisation can be interpreted within the framework of our *Five-Phase Model for a Network-Oriented Exercise Structure*, as described in Sect. ”A Model Structure for Network-Based Therapeutic Exercises”. Though resource-intensive, such individualized approaches are aligned with the principles of network plasticity and offer a rationale for developing flexible, goal-directed therapies.

Importantly, such approaches already exist in neurorehabilitation, and the present framework is intended to integrate and structure these strategies further within a network-oriented perspective in order to provide a foundation that can be developed further and adapted to address patient-specific deficits.

Language impairments (e.g., aphasia after left-hemisphere stroke) can benefit from personalisation strategies such as the use of gesture, demonstration, or the non-paretic limb to support communication and task comprehension [132, 133]. Research indicates that language and gesture are deeply interconnected, sharing overlapping neural substrates [134, 135]. Furthermore, multimodal language processing—which integrates speech and gesture—engages not only left-hemisphere language networks but also right-hemisphere regions involved in spatial and gestural aspects of communication [136, 137].

Impaired motor imagery or planning: Parietal damage can significantly reduce the ability to mentally simulate actions, compromising the internal generation and manipulation of motor representations [79, 138]. Specific regions—such as the left inferior parietal lobule—are particularly implicated in the control and vividness of motor imagery [74]. Right parietal lesions, especially when associated with spatial neglect, may lead to bilateral impairments in imagery performance [76, 139, 140]. Given these potential limitations, it becomes crucial that the therapist’s role involves influencing the patient’s spontaneous imagery, helping to redirect it toward a more accurate and functional motor representation—one that can serve as a foundation for the movement patterns the therapy is designed to promote [141]. In cases where imagery is severely compromised, the therapist may shift the emphasis toward external sensory inputs—visual, tactile, or kinesthetic—as alternative pathways to activate sensorimotor representations and support action planning in line with the neurocognitive approach [72].

Clinically, reduced ability to engage in or report motor imagery, poor task anticipation, or marked deficits in action planning may indicate the need to shift toward externally driven support. Rather than a strict cut-off, this distinction is best conceptualized along a continuum, where the relative integrity of motor imagery, attention, and executive functions informs the degree to which internal versus external strategies are prioritized.

Executive dysfunction may result from damage to prefrontal regions involved in working memory, action selection, and the regulation of goal-directed behaviour [142, 143]. These impairments reflect both localized lesions and broader disruptions to large-scale brain networks that support cognitive control [144, 145]. To address these challenges, interventions such as structured task decomposition, repeated cues, and strategies aimed at enhancing self-monitoring, goal-setting, and regulation of attention and behavior have shown efficacy in scaffolding executive processes and supporting functional learning [146, 147]. These rehabilitation approaches are grounded in contemporary network-based models of brain function, which emphasize the interplay between focal damage and distributed connectivity in shaping cognitive outcomes.

## Robot-assisted network rehabilitation

Robot-assisted therapy has become a standard in rehabilitation clinics over the past decades, with increasing evidence that the outcomes are comparable to those of conventional therapy, while allowing patients to train earlier and at higher intensity [148]. Accordingly, therapy exercises implemented on robotic devices have strongly focused on performing high repetitions of specific kinematic patterns, such as gait cycles or goal-directed reaching, but alternative approaches such as anti-gravity-supported continuous exploration of the workspace have also been investigated [149]. By contrast, exercises that explicitly train proprioceptive discrimination, tactile exploration, or complex cognitive engagement are still rare. As we have shown above, interventions to drive network-level reorganization must overcome the gap between sensory information and cognitive processing.

One promising direction lies in the implementation of exercises inspired by the principles of CMR on robotic systems. While traditional CMR is grounded in direct, embodied interaction between the hand/foot and real objects, robotic systems can physically interact with the patient, enabling the exploration and partial implementation of relevant CMR principles.

In this section, we explore how robotic systems can support both behavioral and neuroimaging research on the mechanisms of network-oriented therapy, as well as its clinical implementation following the three levels of CMR, from (patient) passive to active (from assisted to resisted) movement, and haptic interaction with virtual objects.

The ability of robotic systems to precisely guide patients’ movements under well-controlled and reproducible conditions makes them powerful tools for first-level CMR exercises (physically passive but cognitively active), enabling patients to focus on somatosensory feedback. Introducing a cognitive component in the form of a discrimination task (e.g., using psychophysical methods such as staircase or two-alternative forced-choice procedures) allows researchers to determine somatosensory detection and discrimination thresholds, thereby providing insights into somatosensory impairments [150, 151]. The combination of robotics and psychophysics can also be employed in neuroimaging studies, enabling investigation of the neural networks activated by specific tasks under controlled conditions.

Along these lines, Van de Winckel et al. [108] used an MRI-compatible robot to investigate the neural networks involved in a passive somatosensory discrimination task inspired by CMR, suggesting that different passive discrimination tasks could be used for lesion-specific training following stroke (see Sect. ”Cognitive Multisensory Rehabilitation (CMR) as a Network-Oriented Therapy Approach”). However, the interpretation with solely task-based fMRI findings remains limited, as BOLD signal changes are unable to allow causal inference or distinguish between excitatory and inhibitory neural processes. In another study, functional connectivity changes in bilateral sensorimotor networks related to functional impairment in chronic stroke patients were found following a single session of robot-assisted proprioceptive training [152]. Taken together, these findings highlight the importance of multimodal approaches to more fully characterize the neural mechanisms underlying CMR-based robotic interventions.

To enable passive movement of a patient’s limbs, rehabilitation robots rely on sensors that detect position, orientation and interaction forces and use this data to control their motors. Beyond control, these sensors can also be used to precisely assess motor and somatosensory function of the patient during passive and active movements. As an example, movement kinematics can be used to derive metrics that describe movement features related to those captured by the MESUPES (see Sect. ”Clinical Trials”), such as functional range of motion and movement smoothness during active tasks. In the case of robot-assisted rehabilitation, sensor-based assessments can additionally be used to automatically adapt task difficulty based on the derived metrics, to keep patients motivated and engaged [153], and to enable therapeutic interventions specifically targeting somatosensory function [154].

Active somatosensory perception can be assessed by asking patients to actively reproduce movements that were previously presented to them passively, typically with the ipsilesional arm/hand. This approach can also be extended to active touch. Babadi et al. used a perceptual learning task involving the active detection of small-scale tactile features such as edges to isolate a perceptual learning network using functional connectivity [155]. These features were rendered by a tactile display linked to a sensorized kinematic structure.

Beyond guiding passive and assisting/resisting active movements and providing sensitive and valid assessments, robotic systems can also precisely control the interaction with the patient, by rendering virtual objects such as springs or sponges, as employed in CMR, which the patient can explore haptically [156]. Several groups have proposed robotic systems capable of rendering such virtual environments—e.g., simulating stiffness or viscosity—for haptic interaction during rehabilitation [157]. This allows implementing CMR (or any other type of network-oriented) therapy exercises on robotic systems, taking advantage of their ability to change the properties of virtual objects on the fly, thereby adapting exercise difficulty based on the patient’s performance. When movements in such exercises are actively supported by the robot, the phenomenon of “slacking” should be considered, as assistance may reduce active participation and engagement of the patient [158].

To the best of our knowledge, only one group has directly transferred typical CMR exercises onto a rehabilitation robot [159] and investigated the potential of robot-assisted CMR [160]. The exercises implemented with this robotic device follow the progressively increasing complexity typical of the CMR approach and are aligned with the five-phase model derived from a network-oriented interpretation of the CMR framework (see Sect. ”A Model Structure for Network-Based Therapeutic Exercises”). Thanks to the system’s ability to render virtual object dynamics, the simulated interaction with these virtual objects preserves essential features of genuine haptic interaction—such as the fine modulation of muscle effort and the integration of multisensory inputs—continuously shaped by top-down cognitive processes. This therapy approach has been integrated into the therapy program of the Clinica Hildebrand in Brissago (Switzerland), with the group demonstrating that neurocognitive robot-assisted therapy is not inferior to conventional neurocognitive therapy in an RCT with 32 stroke patients [160].

In rehabilitation centers where the duration of in-patient rehabilitation is short, the full potential of CMR often cannot be exploited, as this approach typically requires more time per exercise compared to simpler, repetitive tasks. However, this limitation can be overcome by providing robot-assisted CMR in an unsupervised manner, allowing patients to independently engage in unsupervised, cognitively engaging therapy during spare time in the clinic or at home. Devittori et al. showed that patients could increase their overall therapy dose in a clinical setting [161]. During two weeks of unsupervised CMR at home, patients trained for 518 min on average, corresponding to a mean increase in therapy dose of 177.5% compared to standard of care [162].

In conclusion, robot-assisted therapy enables standardized, quantifiable, and adaptive interventions. This makes it a powerful tool to support network-based sensorimotor training (see Sect. “Network-Based Rehabilitation”), and to test the effectiveness of network-rehabilitation, both in behavioral studies and in neuroimaging paradigms.

## Serious games and virtual reality in network-based rehabilitation

Virtual reality (VR) uses computer technology to create interactive two- or three-dimensional environments that immerse users in alternative worlds. These may resemble real-life settings or present entirely imaginary or enhanced scenarios. Unlike passive observation, VR enables active participation—users can move around, manipulate objects, experience events, and interact with others in the virtual space.

In VR-assisted rehabilitation, patients engage with these environments and objects through computer screens or head-mounted displays. A related approach, augmented reality (AR), overlays virtual elements onto the real world, allowing users to perceive both simultaneously.

Various efficacy studies have been conducted in stroke patients and summarized in a Cochrane Review on VR and stroke rehabilitation [163]. The 2017 meta-analysis was based on 72 studies involving 2470 people after a stroke using various VR systems, most of which aim to improve walking ability. The authors concluded that “VR was no more beneficial than conventional therapeutic approaches in improving upper limb function”. More recent meta-analyses from 2020 and 2021 found a significant advantage of VR-based training over traditional training for upper extremity motor skills and on executive, visuospatial, and memory functions, but not for gait stability [164, 165].

An insightful study by Maier and colleagues [166] emphasized that not all VR applications are equally effective. Their systematic analysis revealed that only those interventions explicitly designed to leverage neuroscientific principles of plasticity—such as variability, task specificity, multisensory integration, real-time feedback, and cognitive-motor coupling—demonstrated clear therapeutic advantages. In other words, VR is not inherently superior to traditional approaches unless its technical capabilities are aligned with mechanisms known to support network reorganization.

When VR is used strategically, it may support network-based rehabilitation by [6]:

- Providing multisensory feedback (visual, auditory, haptic) that enhances motor learning.
- Enabling cognitive-motor integration, with tasks requiring simultaneous planning, execution, and decision-making [167].
- Supporting adaptive difficulty modulation based on real-time performance metrics.
- Facilitating motivation and autonomy, which are critical for sustained engagement and long-term plasticity [71, 168].

Investigation into the effects of serious gaming technology on brain networks has just begun. A recent study suggests that tactile feedback may support distributed neural network activation in areas related to learning and sensorimotor integration [169] and a recent review concludes that using feedback-based methods enable greater recovery of functional network interactions than standard therapy alone [170].

## Conclusions

We began by emphasizing that stroke is increasingly understood not merely as a focal lesion, but as a disorder of brain networks, affecting both structural and functional connectivity. While these principles are now widely recognized in the theoretical and scientific literature, their systematic translation into clinical practice, particularly in the design and structuring of therapeutic exercises, remains limited or inconsistent. This gap likely reflects the challenges of operationalizing network-based concepts into concrete, task-oriented interventions, rather than a lack of conceptual acceptance among clinicians. Addressing this translational gap represents a key objective of the present framework.

For rehabilitation interventions aimed at enhancing functional ability, the dynamic reorganization of distributed neural systems across the central nervous system represents a foundational element. At the core of this network-oriented approach lies a central insight: motor recovery must leverage the deep interconnection between cognitive, sensory and motor processes, as well as perceptual, emotional, and social processes, all of which contribute to shaping engagement, motivation, and agency in the recovery process. Key components of this interdependence include internal and sensorimotor representations of the body and action, mental preparation (motor imagery), language engagement, and the use of effective learning strategies—all of which collectively contribute to the activation of broad, functionally relevant neural networks.

We have outlined the essential features of a therapy that, in our view, broadly reflects these principles: the neurocognitive approach known as *Cognitive Multisensory Rehabilitation* (CMR), more recently developed into the *Comparison of Actions* (CTA) framework. While not exhaustive, these frameworks illustrate how skill acquisition, underlying capacities, and motivational factors can be integrated to support meaningful, goal-directed behavior within a network-oriented perspective.

Building on this rationale, we proposed a generalizable Model Structure for Network-Based Therapeutic Exercises. This model provides a practical framework for personalizing rehabilitation, allowing task demands to be continuously adapted to the patient’s evolving cognitive and motor profile, in line with principles of optimal challenge and individualized learning.

While we maintain that context-sensitive, cognitively mediated action—based on direct bodily interaction with the world—should remain at the center of rehabilitation, emerging technologies such as robotics and virtual reality offer valuable opportunities to enhance key elements of a network-oriented approach. These technologies can enrich the original framework by improving precision, adaptability, and control of therapeutic interventions, while also increasing the intensity of training. Studies evaluating user experience indicate that both robot-assisted therapy and virtual-reality-based rehabilitation are generally acceptable and feasible for older stroke patients, with high perceived usefulness and manageable usability barriers when appropriate support and design adaptations are in place [171, 172].

In this light, we call for the continued development and rigorous evaluation of network-oriented rehabilitation strategies, including through well-designed clinical trials, to fully assess their therapeutic potential and to help shape the future of neurorehabilitation. Future research should also leverage emerging approaches from artificial intelligence and data science, including AI-informed and adaptive clinical trial designs, to better capture the complexity and individual variability of recovery processes. In addition, integrating mixed-methods approaches that combine quantitative outcomes with qualitative insights may support more bottom-up hypothesis generation and ensure that patient perspectives, often underrepresented in traditional trial designs, are meaningfully incorporated into rehabilitation research. Such integrative approaches may help bridge the gap between mechanistic understanding and real-world rehabilitation practice.

## Data Availability

No datasets were generated or analysed during the current study.

## References

[1] Guggisberg AG, Koch PJ, Hummel FC, Buetefisch CM. Brain networks and their relevance for stroke rehabilitation. Clin Neurophysiol. 2019;130:1098–124. 31082786 10.1016/j.clinph.2019.04.004 PMC6603430

[2] Grefkes C, Fink GR. Connectivity-based approaches in stroke and recovery of function. Lancet Neurol. 2014;13:206–16. 24457190 10.1016/S1474-4422(13)70264-3

[3] Nudo RJ. Recovery after brain injury: mechanisms and principles. Front Hum Neurosci. 2013;7:887. 10.3389/fnhum.2013.00887. 24399951 10.3389/fnhum.2013.00887 PMC3870954

[4] Vahdat S, Lungu O, Cohen-Adad J, Marchand-Pauvert V, Benali H, Doyon J. Simultaneous brain-cervical cord fMRI reveals intrinsic spinal cord plasticity during motor sequence learning. PLoS Biol. 2015;13:e1002186. 10.1371/journal.pbio.1002186. 26125597 10.1371/journal.pbio.1002186 PMC4488354

[5] Wolpaw JR, Thompson AK, Perez MA, Norman SL, Oudega M, Winstein CJ. Neurorehabilitation in the 21st century: new science, new strategies, new expectations. Neurorehabil Neural Repair. 2026. 10.1177/15459683251412309. 41699464 10.1177/15459683251412309 PMC12944605

[6] Verschure PFMJ, Páscoa dos Santos F, Sharma V. Redefining stroke rehabilitation: mobilizing the embodied goal-oriented brain. Curr Opin Neurobiol. 2023;83:102807. 10.1016/j.conb.2023.102807. 10.1016/j.conb.2023.102807 37980804

[7] Bassett DS, Wymbs NF, Porter MA, Mucha PJ, Carlson JM, Grafton ST. Dynamic reconfiguration of human brain networks during learning. Proc Natl Acad Sci U S A. 2011;108:7641–6. 21502525 10.1073/pnas.1018985108 PMC3088578

[8] Cole MW, Bassett DS, Power JD, Braver TS, Petersen SE. Intrinsic and task-evoked network architectures of the human brain. Neuron. 2014;83:238–51. 24991964 10.1016/j.neuron.2014.05.014 PMC4082806

[9] Ptak R, Schnider A, Fellrath J. The dorsal frontoparietal network: a core system for emulated action. Trends Cogn Sci. 2017;21:589–99. 28578977 10.1016/j.tics.2017.05.002

[10] Ptak R, Doganci N, Bourgeois A. From action to cognition: neural reuse, network theory and the emergence of higher cognitive functions. Brain Sci. 2021;11:1652. 34942954 10.3390/brainsci11121652 PMC8699577

[11] Corbetta M, Fitzpatrick SM. Neural rehabilitation: action and manipulation. Neurorehabil Neural Repair. 2011;25(5):3S-5S. 10.1177/1545968311402092. 21613533 10.1177/1545968311402092 PMC4082428

[12] Anderson ML. Neural reuse: a fundamental organizational principle of the brain. Behav Brain Sci. 2010;33:245–66. 20964882 10.1017/S0140525X10000853

[13] Hanakawa T, Immisch I, Toma K, Dimyan MA, Van Gelderen P, Hallett M. Functional properties of brain areas associated with motor execution and imagery. J Neurophysiol. 2003;89:989–1002. 12574475 10.1152/jn.00132.2002

[14] Hardwick RM, Caspers S, Eickhoff SB, Swinnen SP. Neural correlates of action: comparing meta-analyses of imagery, observation, and execution. Neurosci Biobehav Rev. 2018;94:31–44. 30098990 10.1016/j.neubiorev.2018.08.003

[15] Tomasino B, Gremese M. Effects of stimulus type and strategy on mental rotation network: an activation likelihood estimation meta-analysis. Front Hum Neurosci. 2016;9:693. 26779003 10.3389/fnhum.2015.00693 PMC4704562

[16] Zabicki A, De Haas B, Zentgraf K, Stark R, Munzert J, Krüger B. Imagined and executed actions in the human motor system: testing neural similarity between execution and imagery of actions with a multivariate approach. Cereb Cortex. 2017;27:4523–36. 27600847 10.1093/cercor/bhw257

[17] Ganis G, Keenan JP, Kosslyn SM, Pascual-Leone A. Transcranial magnetic stimulation of primary motor cortex affects mental rotation. Cereb Cortex. 2000;10:175–80. 10667985 10.1093/cercor/10.2.175

[18] Tomasino B, Borroni P, Isaja A, Rumiati RI. The role of the primary motor cortex in mental rotation: a TMS study. Cogn Neuropsychol. 2005;22:348–63. 21038255 10.1080/02643290442000185

[19] Desmurget M, Reilly KT, Richard N, Szathmari A, Mottolese C, Sirigu A. Movement intention after parietal cortex stimulation in humans. Science. 2009;324:811–3. 10.1126/science.1169896. 19423830 10.1126/science.1169896

[20] Desmurget M, Sirigu A. Conscious motor intention emerges in the inferior parietal lobule. Curr Opin Neurobiol. 2012;22:1004–11. 22939569 10.1016/j.conb.2012.06.006

[21] Doganci N, Coll SY, Marti E, Ptak R. Anatomical predictors of mental rotation with bodily and non-bodily stimuli: a lesion-symptom study. Neuropsychologia. 2024;193:108775. 38135209 10.1016/j.neuropsychologia.2023.108775

[22] Tian L, Chen H, Heikkinen PP, Liu W, Parviainen T. Spatiotemporal dynamics of activation in motor and language areas suggest a compensatory role of the motor cortex in second language processing. Neurobiol Lang. 2023;4:178–97. 10.1162/nol_a_00093 PMC10205076 37229145

[23] Marti E, Coll SY, Doganci N, Ptak R. Distinct functional connectivity patterns of brain networks involved in motor planning underlie verbal and spatial working memory. Brain Behav. 2025;15:e70376. 40022219 10.1002/brb3.70376 PMC11870840

[24] Bègue I, Elandaloussi Y, Delavari F, Cao H, Moussa-Tooks A, Roser M, et al. The cerebellum and cognitive function: anatomical evidence from a transdiagnostic sample. Cerebellum. 2024;23:1399–410. 38151675 10.1007/s12311-023-01645-y PMC11269336

[25] Schmahmann JD. The cerebellum and cognition. Neurosci Lett. 2019;688:62–75. 29997061 10.1016/j.neulet.2018.07.005

[26] Doron N, Rand D. Is unilateral spatial neglect associated with motor recovery of the affected upper extremity poststroke? Neurorehabil Neural Repair. 2019;33:179–87. 30784364 10.1177/1545968319832606

[27] Corbetta M, Ramsey L, Callejas A, Baldassarre A, Hacker CD, Siegel JS, et al. Common behavioral clusters and subcortical anatomy in stroke. Neuron. 2015;85:927–41. 10.1016/j.neuron.2015.02.027. 25741721 10.1016/j.neuron.2015.02.027 PMC4646844

[28] Hillis AE. Infarct location and dysfunction: what we have learned from lesion-symptom mapping. Neuroscientist. 2014;20:499–510. 10.1177/1073858413514266.24737695

[29] Siegel JS, Seitzman BA, Ramsey LE, Ortega M, Gordon EM, Dosenbach NUF, et al. Disruptions of network connectivity predict impairment in multiple behavioral domains after stroke. Proc Natl Acad Sci U S A. 2016;113:E4367–76. 10.1073/pnas.1521083113. 27402738 10.1073/pnas.1521083113 PMC4968743

[30] Byblow WD, Stinear CM, Barber PA, Petoe MA, Ackerley SJ. Proportional recovery after stroke depends on corticomotor integrity. Ann Neurol. 2015;78:848–59. 10.1002/ana.24472. 26150318 10.1002/ana.24472

[31] Buch ER, Rizk S, Nicolo P, Cohen LG, Schnider A, Guggisberg AG. Predicting motor improvement after stroke with clinical assessment and diffusion tensor imaging. Neurology. 2016;86:1924–5. 10.1212/WNL.0000000000002675. 27164664 10.1212/WNL.0000000000002675 PMC4873682

[32] Feng W, Wang J, Chhatbar PY, Doughty C, Landsittel D, Lioutas VA, et al. Corticospinal tract lesion load: an imaging biomarker for stroke motor outcomes. Ann Neurol. 2015;78:860–70. 10.1002/ana.24510. 26289123 10.1002/ana.24510 PMC4715758

[33] Guggisberg AG, Nicolo P, Cohen LG, Schnider A, Buch ER. Longitudinal structural and functional differences between proportional and poor motor recovery after stroke. Neurorehabil Neural Repair. 2017;31:1029–41. 10.1177/1545968317740634. 29130824 10.1177/1545968317740634 PMC6368856

[34] Thomalla G, Glauche V, Koch MA, Beaulieu C, Weiller C, Röther J. Diffusion tensor imaging detects early Wallerian degeneration of the pyramidal tract after ischemic stroke. Neuroimage. 2004;22:1767–74. 10.1016/j.neuroimage.2004.03.041. 15275932 10.1016/j.neuroimage.2004.03.041

[35] Ma C, Liu A, Li Z, Zhou X, Zhou S. Longitudinal study of diffusion tensor imaging properties of affected corticospinal tracts in acute and chronic hemorrhagic stroke. J Clin Neurosci. 2014;21:1388–92. 10.1016/j.jocn.2013.11.032. 24746110 10.1016/j.jocn.2013.11.032

[36] Thomalla G, Glauche V, Weiller C, Röther J. Time course of Wallerian degeneration after ischaemic stroke revealed by diffusion tensor imaging. J Neurol Neurosurg Psychiatry. 2005;76(2):266–8. 15654048 10.1136/jnnp.2004.046375 PMC1739511

[37] Chen Y, Jiang Y, Kong X, Zhao C, Zhong S, Yang L, et al. Common and unique structural plasticity after left and right hemisphere stroke. J Cereb Blood Flow Metab. 2021;41:3350–64. 10.1177/0271678X211030547. 34415210 10.1177/0271678X211036606 PMC8669287

[38] Von Monakow C. Die Lokalisation im Grosshirn und der Abbau der Funktion durch kortikale Herde. Wiesbaden: J F Bergmann; 1914.

[39] Carrera E, Tononi G. Diaschisis: past, present, future. Brain. 2014;137:2408–22. 10.1093/brain/awu101. 24871646 10.1093/brain/awu101

[40] Maenza C, Good DC, Winstein CJ, Wagstaff DA, Sainburg RL. Functional deficits in the less-impaired arm of stroke survivors depend on hemisphere of damage and extent of paretic arm impairment. Neurorehabil Neural Repair. 2020;34:39–50. 10.1177/1545968319875951 10.1177/1545968319875951 PMC6954970 31538852

[41] Maenza C, Winstein CJ, Murphy TE, Kitchen NM, Tanaka J, Yuk J, et al. Targeted remediation of the ipsilesional arm in chronic stroke: a randomized clinical trial. JAMA Neurol. 2026;83:223–30. 10.1001/jamaneurol.2025.5496. 41627841 10.1001/jamaneurol.2025.5496 PMC12865693

[42] Buch ER, ModirShanechi A, Fourkas AD, Weber C, Birbaumer N, Cohen LG. Parietofrontal integrity determines neural modulation associated with grasping imagery after stroke. Brain. 2012;135:596–614. 10.1093/brain/awr331. 22232595 10.1093/brain/awr331 PMC3286199

[43] Nicolo P, Rizk S, Magnin C, Di Pietro M, Schnider A, Guggisberg AG. Coherent neural oscillations predict future motor and language improvement after stroke. Brain. 2015;138:3048–60. 10.1093/brain/awv200. 26163304 10.1093/brain/awv200

[44] Wang L, Yu C, Chen H, Qin W, He Y, Fan F, et al. Dynamic functional reorganization of the motor execution network after stroke. Brain. 2010;133:1224–38. 10.1093/brain/awq043. 20354002 10.1093/brain/awq043

[45] Xu J, Branscheidt M, Schambra H, Steiner L, Widmer M, Diedrichsen J, et al. Rethinking interhemispheric imbalance as a target for stroke neurorehabilitation. Ann Neurol. 2019;85:502–13. 10.1002/ana.25452. 30805956 10.1002/ana.25452 PMC7374229

[46] Wolpaw JR, Kamesar A. Heksor: the central nervous system substrate of an adaptive behaviour. J Physiol. 2022;600:3423–52. 10.1113/JP283291. 35771667 10.1113/JP283291 PMC9545119

[47] Baier B, Karnath H-O. Tight link between our sense of limb ownership and self-awareness of actions. Stroke. 2008;39:486–8. 10.1161/STROKEAHA.107.495606. 18162622 10.1161/STROKEAHA.107.495606

[48] Braun N, Debener S, Spychala N, Bongartz E, Sörös P, Müller HH, et al. The senses of agency and ownership: a review. Front Psychol. 2018;9:535. 29713301 10.3389/fpsyg.2018.00535 PMC5911504

[49] Pia L, Neppi-Modona M, Ricci R, Berti A. The anatomy of anosognosia for hemiplegia: a meta-analysis. Cortex. 2004;40:367–77. 15156794 10.1016/s0010-9452(08)70131-x

[50] Jehkonen M, Laihosalo M, Kettunen JE. Anosognosia after stroke: assessment, occurrence, subtypes and impact on functional outcome reviewed. Cortex. 2006;42:495–503. 10.1016/S0010-9452(08)70382-2. 17022776 10.1111/j.1600-0404.2006.00723.x

[51] Varghese R, Gordon JE, Sainburg RL, Winstein CJ, Schweighofer N. Adaptive control is reversed between hands after left hemisphere stroke and lost following right hemisphere stroke. Proc Natl Acad Sci USA. 2023. 10.1073/pnas.2212726120. 36716370 10.1073/pnas.2212726120 PMC9963612

[52] Calabrò RS, Quartarone A. Neurorehabilitation as Network Perturbation: Shaping Neuroplasticity with Robotics, Virtual Reality, and Neuromodulation. Biomedicines. 2026;14:411 10.3390/biomedicines14020411. 10.3390/biomedicines14020411 PMC12937845 41751310

[53] Li W, George P, Azadian MM, Ning M, Dhand A, Cramer SC, et al. Changing genes, cells and networks to reprogram the brain after stroke. Nat Neurosci. 2025;28:1130–45. 10.1038/s41593-025-01981-8. 40456908 10.1038/s41593-025-01981-8

[54] Tsay JS, Kim HE, McDougle SD, Taylor JA, Haith A, Avraham G, et al. Fundamental processes in sensorimotor learning: reasoning, refinement, and retrieval. eLife. 2024;12:e91839. 10.7554/eLife.91839 PMC11293869 39087986

[55] Perfetti C. La rieducazione motoria dell’emiplegico. Milano: Ghedini Editore; 1979.

[56] Perfetti C. Condotte terapeutiche per la rieducazione motoria dell’emiplegico. Milano: Ghedini Editore; 1982.

[57] Perfetti C, Noccioli W. Per una pragmatica del movimento. Riabilitazione Apprendimento. 1986;2:119–32.

[58] Perfetti C. Condotte terapeutiche per la rieducazione motoria dell’emiplegico. Milano: Ghedini Editore; 1987.

[59] Perfetti C, Pieroni A. La logica dell’esercizio. Napoli: Idelson; 1992.

[60] Argüelles V, Cracchiolo M, De Patre D, Ferrer Davesa M, Nani C, Rigoni M, Romeo F, Wopfner S. La teoria neurocognitiva secondo il confronto tra azioni. Vol. I. Padua: Piccin-Nuova Libraria. 2021.

[61] Perfetti C, Panté F, Rizzello C. Dall’esercizio terapeutico conoscitivo al Confronto tra Azioni: quali implicazioni riabilitative? Riv Riabil Neurocognitiva. 2014;2:4–15.

[62] Mustile M, Kourtis D, Edwards MG, Donaldson DI, Ietswaart M. Neural correlates of motor imagery and execution in real-world dynamic behavior: evidence for similarities and differences. Front Hum Neurosci. 2024;18:1412307. 10.3389/fnhum.2024.1412307. 38974480 10.3389/fnhum.2024.1412307 PMC11224467

[63] Tong Y, Yang Q, Chen S, Huang J, Ma S, Xu Q, et al. Applications of motor imagery to rehabilitation: a review. Aging Dis. 2017;8:364–70. 28580191 10.14336/AD.2016.1012 PMC5440115

[64] Wang W, Yu X, Wu Y, Wang Y, Zhang Y, Wang J. Reorganization of functional brain networks during motor imagery and execution in stroke patients. Neural Regen Res. 2016;11:1278–84.27651776

[65] Panté F. L’utilizzo dell’immagine motoria. Riabilitazione Apprendimento. 1997;17:127–38.

[66] Perfetti C, Rossetto F. L’immagine motoria come elemento dell’esercizio terapeutico. Riabilitazione Apprendimento. 1997;17:109–16.

[67] Perfetti C. Immagine motoria, rappresentazione mentale. Riabilitazione Cognitiva. 2000;1:13–29.

[68] Farah MJ. The neurological basis of mental imagery: a componential analysis. Cognition. 1984;18(1–3):245–72. 10.1016/0010-0277(84)90026. 6396031 10.1016/0010-0277(84)90026-x

[69] Hall C, Buckolz E, Fishburne G. Searching for a relationship between imagery ability and memory of movements. J Mot Behav. 1989;17:89–100.

[70] Kosslyn SM. Les images mentales. La Recherche. 1980;108:156–63.

[71] Wulf G, Lewthwaite R. Optimizing performance through intrinsic motivation and attention for learning: the OPTIMAL theory of motor learning. Psychon Bull Rev. 2016;23:1382–414. 10.3758/s13423-015-0999-9. 26833314 10.3758/s13423-015-0999-9

[72] Perfetti C. Die motorische Imagination. In: Rehabilitieren mit Gehirn. München: Pflaum Verlag; 2007.

[73] Decety J, Jeannerod M, Prablanc C. The neurophysiological basis of motor imagery. Behav Brain Res. 1994;52:13–22. 10.1016/0166-4328(94)90128-7. 10.1016/0166-4328(95)00225-1 8762158

[74] Kraeutner S, El-Serafi M, Lee JW, Boe SG. Disruption of motor imagery performance following inhibition of the left inferior parietal lobe. Neuropsychologia. 2019;127:106–12. 30807756 10.1016/j.neuropsychologia.2019.02.016

[75] Malouin F, Richards CL, Jackson PL, Doyon J, Durand A. The kinesthetic and visual imagery questionnaire (KVIQ) for assessing motor imagery in persons with physical disabilities: a reliability and construct validity study. J Neurol Phys Ther. 2007;31:20–9. 10.1097/01.NPT.0000281945.03186.62. 17419886 10.1097/01.npt.0000260567.24122.64

[76] Sirigu A, Duhamel JR, Cohen L, Pillon B, Dubois B, Agid Y. The mental representation of hand movements after parietal cortex damage. Science. 1996;273:1564–8. 10.1126/science.8943200. 8703221 10.1126/science.273.5281.1564

[77] McInnes K, Friesen C, Boe S. Specific Brain Lesions Impair Explicit Motor Imagery Ability: A Systematic Review of the Evidence. Arch Phys Med Rehabil. 2016;97(3):478–e4891. 26254950 10.1016/j.apmr.2015.07.012

[78] Hétu S, Gregoire M, Saimpont A, Coll MP, Eugène F, Michon PE, et al. The neural network of motor imagery: an ALE meta-analysis. Neurosci Biobehav Rev. 2013;37:930–49. 10.1016/j.neubiorev.2013.03.017. 23583615 10.1016/j.neubiorev.2013.03.017

[79] Oostra KM, Van Bladel A, Vanhoonacker ACL, Vingerhoets G. Damage to fronto-parietal networks impairs motor imagery ability after stroke. Front Behav Neurosci. 2016;10:5. 10.3389/fnbeh.2016.00005. 26869894 10.3389/fnbeh.2016.00005 PMC4740776

[80] Kapetaniou GE, Moisa M, Ruff CC, Tobler PN, Soutschek A. Frontopolar cortex interacts with dorsolateral prefrontal cortex to causally guide metacognition. Hum Brain Mapp. 2025;46:e70146. 39878207 10.1002/hbm.70146 PMC11775761

[81] Qiu L, Su J, Ni Y, Bai Y, Zhang X, Li X, et al. The neural system of metacognition accompanying decision-making in the prefrontal cortex. PLoS Biol. 2018;16:e2004037. 10.1371/journal.pbio.2004037. 29684004 10.1371/journal.pbio.2004037 PMC5933819

[82] Rahneva D, Neeb DE, Riddle C, Larson AL, D’Esposito M. Causal evidence for frontal cortex organization for perceptual decision making. Proc Natl Acad Sci U S A. 2016;113:6059–64. 27162349 10.1073/pnas.1522551113 PMC4889369

[83] Alderson-Day B, Fernyhough C. Inner speech: development, cognitive functions, phenomenology, and neurobiology. Psychol Bull. 2015;141:931–65. 26011789 10.1037/bul0000021 PMC4538954

[84] Langland-Hassan P. Inner speech and cognitive performance: evidence for a relationship. In: Vicente A, Martínez-Manrique F, editors. Inner speech: new voices. Oxford: Oxford University Press; 2014. p. 166–92.

[85] Leech KA, Roemmich RT, Gordon J, Reisman DS, Cherry-Allen KM. Updates in motor learning: implications for physical therapist practice and education. Phys Ther. 2022;102:pzac015. 34718787 10.1093/ptj/pzab250 PMC8793168

[86] Seidler RD, Bo J, Anguera JA. Neurocognitive contributions to motor skill learning: the role of working memory. J Mot Behav. 2012;44:445–53. 10.1080/00222895.2012.672348. 23237467 10.1080/00222895.2012.672348 PMC3534841

[87] Spampinato D, Celnik P. Multiple motor learning processes in humans: defining their neurophysiological bases. Neuroscientist. 2021;27:246–67. 32713291 10.1177/1073858420939552 PMC8151555

[88] Lindsay RS, Spittle S, Spittle M. Considering the need for movement variability in motor imagery training: implications for sport and rehabilitation. Front Psychol. 2023;14:1178632. 10.3389/fpsyg.2023.1178632. 37251018 10.3389/fpsyg.2023.1178632 PMC10213205

[89] Lindsay RS, Komar J, Chow JY, Larkin P, Spittle M. Different pedagogical approaches to motor imagery both demonstrate individualized movement patterns to achieve improved performance outcomes when learning a complex motor skill. PLoS ONE. 2023;18:e0282647. 10.1371/journal.pone.0282647. 38019823 10.1371/journal.pone.0282647 PMC10686457

[90] Sato Y. Effects of motor imagery training on generalization and retention for different task difficulties. Front Hum Neurosci. 2024;18:1459987. 10.3389/fnhum.2024.1459987. 39479228 10.3389/fnhum.2024.1459987 PMC11521821

[91] Boutin A, Blandin Y, Massen C, Heuer H, Badets A. Conscious awareness of action potentiates sensorimotor learning. Cognition. 2014;133:1–9. 10.1016/j.cognition.2014.05.012. 24954450 10.1016/j.cognition.2014.05.012

[92] Doyon J, Benali H. Reorganization and plasticity in the adult brain during learning of motor skills. Curr Opin Neurobiol. 2005;15:161–7. 10.1016/j.conb.2005.03.004. 15831397 10.1016/j.conb.2005.03.004

[93] Shiffrin RM, Schneider W. Controlled and automatic human information processing: II. Perceptual learning, automatic attending, and a general theory. Psychol Rev. 1977;84:127–90. 10.1037/0033-295X.84.2.127.

[94] Caspers J, Mathys C, Hoffstaedter F, Südmeyer M, Cieslik EC, Rubbert C, et al. Differential functional connectivity alterations of two subdivisions within the right dlPFC in Parkinson’s disease. Front Hum Neurosci. 2017;11:288. 10.3389/fnhum.2017.00288. 28611616 10.3389/fnhum.2017.00288 PMC5447710

[95] Inman CS, James GA, Hamann S, Rajendra JK, Pagnoni G, Butler AJ. Altered resting-state effective connectivity of fronto-parietal motor control systems on the primary motor network following stroke. Neuroimage. 2012;59:227–37. 10.1016/j.neuroimage.2011.07.085. 21839174 10.1016/j.neuroimage.2011.07.083 PMC3195990

[96] Del-Cuvillo-Yges M, Escudero A, Moreta de Esteban P, López-Marcos JJ, Martín-Casas P. Systematic review on the effectiveness of cognitive multisensory rehabilitation. An Sist Sanit Navar. 2022;45:e1013. 10.23938/ASSN.1013 PMC10065038 36408570

[97] Kim KH, Jang SH. Effects of cognitive sensory motor training on lower extremity muscle strength and balance in post-stroke patients: a randomized controlled study. Clin Pract. 2021;11:640–9. 10.3390/clinpract11030079. 34563008 10.3390/clinpract11030079 PMC8482150

[98] Dominguez-Ferraz D, da Silva-Ribeiro NM, de Matos-Pinheiro I, Pedreira-da FÉ. Eficacia del método Perfetti en el tratamiento de secuelas del accidente cerebrovascular: una revisión sistemática. Cuest Fisioter. 2014;43:196–205.

[99] Cardile D, Lo Buono V, Corallo F, Cammaroto S, Formica C, Quartarone A, et al. The importance of recovering body awareness in post-stroke rehabilitation: insights from clinical case reports. Front Neurol. 2024;15:1467181. 10.3389/fneur.2024.1467181. 39726757 10.3389/fneur.2024.1467181 PMC11669554

[100] Lee S, Bae S, Jeon D, Kim KY. The effects of cognitive exercise therapy on chronic stroke patients’ upper limb functions, activities of daily living and quality of life. J Phys Ther Sci. 2015;27:2787–91. 26504294 10.1589/jpts.27.2787 PMC4616095

[101] Sallés L, Martín-Casas P, Gironès X, Durà MJ, Lafuente JV, Perfetti C. A neurocognitive approach for recovering upper extremity movement following subacute stroke: a randomized controlled pilot study. J Phys Ther Sci. 2017;29:665–72. 28533607 10.1589/jpts.29.665 PMC5430270

[102] Huai Y, Yang W, Lv Y, Wang K, Zhou H, Lu Y, et al. Enriched rehabilitation on brain functional connectivity. Front Neurol. 2025;15:1503737. 10.3389/fneur.2024.1503737. 39845930 10.3389/fneur.2024.1503737 PMC11752906

[103] Krakauer JW, Carmichael ST. Broken movement: the neurobiology of motor recovery after stroke. Cambridge (MA): MIT Press; 2017.

[104] Chen JL, Schlaug G, Small SL. The future of stroke rehabilitation: a neuroanatomically informed, personalized, and biomarker-guided approach. Neurorehabil Neural Repair. 2020;34:751–64.

[105] Kwakkel G, van Wegen E, Burridge JH, Winstein CJ, van Dokkum L, Alt Murphy M, et al. Standardized measurement of quality of upper limb movement after stroke: consensus-based core recommendations from the second stroke recovery and rehabilitation roundtable. Int J Stroke. 2019;14:783–91. 10.1177/1747493019873519. 31510885 10.1177/1747493019873519

[106] Van de Winckel A, Feys H, van der Knaap S, et al. Can quality of movement be measured? Rasch analysis and inter-rater reliability of the Motor Evaluation Scale for Upper Extremity in Stroke Patients (MESUPES). Clin Rehabil. 2006;20:871–84. . 10.1177/0269215506072181 17008339 10.1177/0269215506072181

[107] Van de Winckel A, De Patre D, Rigoni M, et al. Exploratory study of how Cognitive Multisensory Rehabilitation restores parietal operculum connectivity and improves upper limb movements in chronic stroke. Sci Rep. 2020;10:20278. 10.1038/s41598-020-77361-0. 33219267 10.1038/s41598-020-77272-y PMC7680110

[108] Van de Winckel A, De Weerdt W, Feys H, et al. Passive somatosensory discrimination: training-induced cortical plasticity in adults with chronic stroke. Neuroimage. 2005;26:441–53. 15907302 10.1016/j.neuroimage.2005.01.058

[109] Van de Winckel A, Feys H, De Weerdt W, et al. Frontoparietal involvement in passively guided shape and length discrimination. Exp Brain Res. 2012;220:179–89. 22648204 10.1007/s00221-012-3128-2

[110] Van de Winckel A, Klingels K, Bruyninckx H, et al. Brain activation differences in cerebral palsy. Res Dev Disabil. 2013a;34:183–97. 22940170 10.1016/j.ridd.2012.07.030

[111] Van de Winckel A, Klingels K, Wenderoth N, et al. Somatosensory discrimination in children. Res Dev Disabil. 2013b;34:1710–20. 23500165 10.1016/j.ridd.2013.02.017

[112] Liew SL, Cotton RJ, Burdet E, Bermúdez i Badia S, Celnik P, Cole JH, et al. Collaborative AI for precision neurorehabilitation: a roadmap. J Neuroeng Rehabil. 2025. 10.1186/s12984-025-01810-w. 10.1186/s12984-025-01810-w PMC12751918 41310676

[113] Ye D, Luo H, Winstein C, Schweighofer N. Towards AI-based precision rehabilitation via contextual model-based reinforcement learning. J Neuroeng Rehabil. 2025;22:263. 10.1186/s12984-025-01771-0. 41419889 10.1186/s12984-025-01771-0 PMC12717728

[114] Peigneux P, Van der Linden M, Garraux G, Laureys S, Degueldre C, Aerts J, et al. Imaging a cognitive model of apraxia: the neural substrate of gesture-specific cognitive processes. Hum Brain Mapp. 2004;21:119–42. 14755833 10.1002/hbm.10161 PMC6872064

[115] Goldenberg G. Apraxia: the cognitive side of motor control. Oxford: Oxford University Press; 2013.

[116] Schack T, Frank C. Mental representation and the cognitive architecture of skilled action. Rev Philos Psychol. 2021;12:527–46. 10.1007/s13164-020-00485-7.

[117] Gippert M, Pei-Cheng S, Heed T, Howard IS, Idaji MJ, Villringer A, et al. Motor imagery enhances performance beyond the imagined action. Proc Natl Acad Sci U S A. 2025;122:e2423642122. 10.1073/pnas.2423642122. 40359042 10.1073/pnas.2423642122 PMC12107166

[118] Elsner B, Hommel B. Effect anticipation and action control. J Exp Psychol Hum Percept Perform. 2001;27:229–40. 10.1037/0096-1523.27.1.229. 11248937 10.1037//0096-1523.27.1.229

[119] Hommel B, Müsseler J, Aschersleben G, Prinz W. The theory of event coding (TEC): a framework for perception and action planning. Behav Brain Sci. 2001;24:849–78. 10.1017/S0140525X01000103. 12239891 10.1017/s0140525x01000103

[120] Jeannerod M. Neural simulation of action: a unifying mechanism for motor cognition. Neuroimage. 2001;14:S103–9. 10.1006/nimg.2001.0832. 11373140 10.1006/nimg.2001.0832

[121] Rushworth MFS, Kolling N, Sallet J, Mars RB. Valuation and decision-making in frontal cortex: one or many serial or parallel systems? Curr Opin Neurobiol. 2012;22:946–55. 10.1016/j.conb.2012.04.011. 22572389 10.1016/j.conb.2012.04.011

[122] Schultz W. Dopamine reward prediction error coding. Dialogues Clin Neurosci. 2016;18:23–32. 27069377 10.31887/DCNS.2016.18.1/wschultz PMC4826767

[123] Shenhav A, Botvinick MM, Cohen JD. The expected value of control: an integrative theory of anterior cingulate cortex function. Neuron. 2013;79:217–40. 10.1016/j.neuron.2013.07.007. 23889930 10.1016/j.neuron.2013.07.007 PMC3767969

[124] Shadmehr R, Krakauer JW. A computational neuroanatomy for motor control. Exp Brain Res. 2008;185:359–81. 10.1007/s00221-008-1280-5. 18251019 10.1007/s00221-008-1280-5 PMC2553854

[125] Wolpert DM, Ghahramani Z. Computational principles of movement neuroscience. Nat Neurosci. 2000;3(Suppl):1212–7. 10.1038/81497. 11127840 10.1038/81497

[126] Arbuckle SA, Pruszynski JA, Diedrichsen J. Mapping the integration of sensory information across fingers in human sensorimotor cortex. J Neurosci. 2022;42:5173–85. 10.1523/JNEUROSCI.2152-21.2022. 35606141 10.1523/JNEUROSCI.2152-21.2022 PMC9236287

[127] Krakauer JW, Hadjiosif AM, Xu J, Wong AL, Haith AM. Motor learning. Nat Rev Neurosci. 2019;20:389–403. 10.1038/s41583-019-0150-3.

[128] Shadmehr R, Smith MA, Krakauer JW. Error correction, sensory prediction, and adaptation in motor control. Annu Rev Neurosci. 2010;33:89–108. 10.1146/annurev-neuro-060909-153135. 20367317 10.1146/annurev-neuro-060909-153135

[129] Ackerley R, Borich M, Oddo CM, Ionta S. Insights and perspectives on sensory-motor integration and rehabilitation. Multisens Res. 2016. 10.1163/22134808-00002530.

[130] Winstein CJ, Lewthwaite R, Blanton SR, Wolf LB, Wishart L. Infusing motor learning research into neurorehabilitation practice: a historical perspective with case exemplar from the accelerated skill acquisition program. J Neurol Phys Ther. 2014;38:190–200. 10.1097/NPT.0000000000000046. 24828523 10.1097/NPT.0000000000000046 PMC5348298

[131] Guadagnoli MA, Lee TD. Challenge point: a framework for conceptualizing the effects of various practice conditions in motor learning. J Mot Behav. 2004;36:212–24. 15130871 10.3200/JMBR.36.2.212-224

[132] Clough S, Duff MC. The role of gesture in communication and cognition. Front Hum Neurosci. 2020;14:323. 10.3389/fnhum.2020.00323. 32903691 10.3389/fnhum.2020.00323 PMC7438760

[133] Cocks N, Byrne S, Pritchard M, Morgan G, Dipper L. Integration of speech and gesture in aphasia. Int J Lang Commun Disord. 2018;53:584–91. 29411476 10.1111/1460-6984.12372

[134] Bellugi U. Language, spatial cognition, and brain organization. In: Neuropsychology: the neuronal basis of cognitive function. Vol. 2. New York: Thieme Medical Publishers; 1992. p. 207–22.

[135] Willems RM, Hagoort P. Neural evidence for interplay between language and action. Brain Lang. 2007;101:278–89. 17416411 10.1016/j.bandl.2007.03.004

[136] Emmorey K, Damasio H, McCullough S, Grabowski T, Ponto LL, Hichwa RD, et al. Neural systems underlying spatial language in American Sign Language. Neuroimage. 2002;17:812–24. 12377156

[137] Floegel M, Fuchs S, Kell CA. Differential contributions of the two cerebral hemispheres to temporal and spectral speech feedback control. Nat Commun. 2020;11:2839. 10.1038/s41467-020-16743-2. 32503986 10.1038/s41467-020-16743-2 PMC7275068

[138] Sharma N, Pomeroy VM, Baron JC. Motor imagery: a backdoor to the motor system after stroke? Stroke. 2006;37:1941–52. 16741183 10.1161/01.STR.0000226902.43357.fc

[139] Danckert J, Ferber S, Doherty T, Steinmetz H, Nicolle D, Goodale MA. Selective, non-lateralized impairment of motor imagery. Neurocase. 2002;8:194–204. 12119314 10.1093/neucas/8.3.194

[140] Sirigu A, Daprati E, Pradat-Diehl P, Franck N, Jeannerod M. Perception of self-generated movement. Brain. 1999;122:1867–74. 10506089 10.1093/brain/122.10.1867

[141] Conti FM. Motorische Imagination in der Neurorehabilitation. Neurology.ch. 2011;4.

[142] Friedman NP, Robbins TW. The role of prefrontal cortex in cognitive control and executive function. Neuropsychopharmacology. 2022;47:72–89. 10.1038/s41386-021-01132-0. 34408280 10.1038/s41386-021-01132-0 PMC8617292

[143] Stuss DT, Levine B. Adult clinical neuropsychology: lessons from studies of the frontal lobes. Annu Rev Psychol. 2002;53:401–33. 10.1146/annurev.psych.53.100901.135220. 11752491 10.1146/annurev.psych.53.100901.135220

[144] Lim J, Lee J, Woo CW. Post-stroke cognitive impairment: pathophysiological insights into brain disconnectome from advanced neuroimaging analysis techniques. J Stroke. 2021;23:297–311. 10.5853/jos.2021.02376. 34649376 10.5853/jos.2021.02376 PMC8521255

[145] Miller EK, Cohen JD. An integrative theory of prefrontal cortex function. Annu Rev Neurosci. 2001;24:167–202. 10.1146/annurev.neuro.24.1.167. 11283309 10.1146/annurev.neuro.24.1.167

[146] Cicerone KD, Goldin Y, Ganci K, Rosenbaum A, Wethe JV, Langenbahn DM, et al. Evidence-based cognitive rehabilitation: systematic review of the literature from 2009 through 2014. Arch Phys Med Rehabil. 2019;100:1515–33. 30926291 10.1016/j.apmr.2019.02.011

[147] Levine B, Schweizer TA, O’Connor C, Turner G, Gillingham S, Stuss DT, et al. Rehabilitation of executive functioning in patients with frontal lobe brain damage with goal management training. Front Hum Neurosci. 2011;5:9. 10.3389/fnhum.2011.00009. 21369362 10.3389/fnhum.2011.00009 PMC3043269

[148] Gassert R, Dietz V. Rehabilitation robots for the treatment of sensorimotor deficits: a neurophysiological perspective. J Neuroeng Rehabil. 2018;15:46. 29866106 10.1186/s12984-018-0383-x PMC5987585

[149] Krakauer JW, Kitago T, Goldsmith J, Ahmad O, Roy P, Stein J, et al. Comparing a novel neuroanimation experience to conventional therapy for high-dose intensive upper-limb training in subacute stroke: the SMARTS2 randomized trial. Neurorehabil Neural Repair. 2021;35(5):393–405. 33745372 10.1177/15459683211000730

[150] Zbytniewska-Mégret M, Salzmann C, Kanzler CM, Hassa T, Gassert R, Lambercy O, et al. The evolution of hand proprioceptive and motor impairments in the sub-acute phase after stroke. Neurorehabil Neural Repair. 2023;37:823–36. 37953595 10.1177/15459683231207355 PMC10685702

[151] Semrau JA, Herter TM, Scott SH, Dukelow SP. Examining differences in patterns of sensory and motor recovery after stroke with robotics. Stroke. 2015;46:3459–64. 26542695 10.1161/STROKEAHA.115.010750

[152] Vahdat S, Darainy M, Thiel A, Ostry DJ. A single session of robot-controlled proprioceptive training modulates functional connectivity of sensory motor networks and improves reaching accuracy in chronic stroke. Neurorehabil Neural Repair. 2019;33:70–81. 30595082 10.1177/1545968318818902 PMC6389407

[153] Metzger JC, Lambercy O, Califfi A, Dinacci D, Petrillo C, Rossi P, et al. Assessment-driven selection and adaptation of exercise difficulty in robot-assisted therapy: a pilot study with a hand rehabilitation robot. J Neuroeng Rehabil. 2014;11:154. 10.1186/1743-0003-11-154. 25399249 10.1186/1743-0003-11-154 PMC4273449

[154] Yeh IL, Holst-Wolf J, Elangovan N, Cuppone AV, Lakshminarayan K, Cappello L, et al. Effects of a robot-aided somatosensory training on proprioception and motor function in stroke survivors. J Neuroeng Rehabil. 2021;18:77. 33971912 10.1186/s12984-021-00871-x PMC8112068

[155] Babadi S, Gassert R, Hayward V, Piccirelli M, Kollias S, Milner TE. Brain network for small-scale features in active touch. Neuroimage Rep. 2022;2:100123. 40567573 10.1016/j.ynirp.2022.100123 PMC12172874

[156] Metzger JC, Lambercy O, Gassert R. Performance comparison of interaction control strategies on a hand rehabilitation robot. In: Proc IEEE Int Conf Rehabil Robot (ICORR). 2015;846–51.

[157] Rätz R, Conti F, Thaler I, Müri RM, Marchal-Crespo L. Enhancing stroke rehabilitation with whole-hand haptic rendering: development and clinical usability evaluation of a novel upper-limb rehabilitation device. J Neuroeng Rehabil. 2024;21:172. 39334423 10.1186/s12984-024-01439-1 PMC11437669

[158] Marchal-Crespo L, McHughen S, Cramer SC, Reinkensmeyer DJ. The effect of haptic guidance, aging, and initial skill level on motor learning of a steering task. Exp Brain Res. 2010;201:209–20. 19820920 10.1007/s00221-009-2026-8 PMC2832903

[159] Metzger JC, Lambercy O, Califfi A, Conti FM, Gassert R. Neurocognitive robot-assisted therapy of hand function. IEEE Trans Haptics. 2014;7:140–9. 24968378 10.1109/TOH.2013.72

[160] Ranzani R, Lambercy O, Metzger JC, Califfi A, Regazzi S, Dinacci D, et al. Neurocognitive robot-assisted rehabilitation of hand function: a randomized controlled trial on motor recovery in subacute stroke. J Neuroeng Rehabil. 2020;17:115. 32831097 10.1186/s12984-020-00746-7 PMC7444058

[161] Devittori G, Dinacci D, Romiti D, Califfi A, Petrillo C, Rossi P, et al. Unsupervised robot-assisted rehabilitation after stroke: feasibility, effect on therapy dose, and user experience. J Neuroeng Rehabil. 2024;21:52. 38594727 10.1186/s12984-024-01347-4 PMC11005116

[162] Devittori G, Dinacci D, Petrillo C, Rossi P, Gassert R, Lambercy O. Unsupervised robot-assisted therapy at home after stroke: a pilot feasibility study. In: Proc IEEE Int Conf Rehabil Robot (ICORR). 2025;166–71. 10.1109/ICORR66766.2025.11062934 40644292

[163] Laver KE, Lange B, George S, Deutsch JE, Saposnik G, Crotty M. Virtual reality for stroke rehabilitation. Cochrane Database Syst Rev. 2017;11:CD008349. 10.1002/14651858.CD008349.pub4. 29156493 10.1002/14651858.CD008349.pub4 PMC6485957

[164] Rutkowski J. Rehabilitated with exoskeletons: a new opportunity for patients after spinal cord injury? J Rehabil Med. 2020;52:jrm00121. 10.2340/16501977-2755. 33073855 10.2340/16501977-2755

[165] Zhang Y. Impact of Virtual Reality-Based Therapies on Cognition and Mental Health of Stroke Patients: Systematic Review and Meta-analysis. J Med Internet Res. 2021;23:e31007. 10.2196/31007. 34787571 10.2196/31007 PMC8663637

[166] Maier M, Ballester BR, Verschure PFMJ. Principles of neurorehabilitation after stroke based on motor learning and brain plasticity mechanisms. Neurorehabil Neural Repair. 2019;33:112–29. 10.1177/1545968318820169. 30700224 10.1177/1545968318820169 PMC6376608

[167] Morone G, Paolucci S, Iosa M. In what direction is the clinical rehabilitation of stroke patients going? An overview. Front Neurol. 2019;10:374. 10.3389/fneur.2019.00374.

[168] Lohse K, Shirzad N, Verster A, Hodges N, Van der Loos HFM. Video games and rehabilitation: using design principles to enhance engagement in physical therapy. J Neurol Phys Ther. 2013;37:166–75. 10.1097/NPT.0000000000000017. 24232363 10.1097/NPT.0000000000000017

[169] Chen L, Meng F, Huo C, Shao G, Pan G, Zhang X, Zhang S, Li Z. Effects of tactile feedback in post-stroke hand rehabilitation on functional connectivity and cortical activation: an fNIRS study. Biomed Opt Express. 2025;16(2):643–56. 10.1364/BOE.541820 39958859 10.1364/BOE.541820 PMC11828458

[170] Kuipers JA, Hoffman NH, Carrick FR, Jemni M. Reconnecting brain networks after stroke: a scoping review of conventional, neuromodulatory, and feedback-driven rehabilitation approaches. Brain Sci. 2025;15:1217. 10.3390/brainsci15111217. 41300224 10.3390/brainsci15111217 PMC12651463

[171] Ding K, Ma Y, Zhang L, Gu Y, Pan H, Gu Z-E, et al. Patient-centered insights into virtual reality rehabilitation for stroke: a systematic review and qualitative meta-synthesis. J Neuroeng Rehabil. 2025;22:124. 10.1186/s12984-025-01641-9. 40452007 10.1186/s12984-025-01641-9 PMC12128322

[172] Sloan-Garza E, Wilson J, Techan A, Joseph S, Weerasinghe H, Kim Y, et al. Feasibility and acceptability of virtual reality for rehabilitation after acute stroke in patients of various ages. Am J Crit Care. 2026;35:62–7. 10.4037/ajcc2026529. 41475781 10.4037/ajcc2026529

